# Risk and Prediction of Sudden Cardiac Death and Ventricular Arrhythmias for Patients with Atrial Fibrillation – A Nationwide Cohort Study

**DOI:** 10.1038/srep46445

**Published:** 2017-04-19

**Authors:** Tze-Fan Chao, Chia-Jen Liu, Ta-Chuan Tuan, Su-Jung Chen, Tzeng-Ji Chen, Gregory Y. H. Lip, Shih-Ann Chen

**Affiliations:** 1 Division of Cardiology, Department of Medicine, Taipei Veterans General Hospital, Taipei, Taiwan; 2 Institute of Clinical Medicine, and Cardiovascular Research Center, National Yang-Ming University, Taipei, Taiwan; 3 Division of Hematology and Oncology, Department of Medicine, Taipei Veterans General Hospital, Taipei, Taiwan; 4 Institute of Public Health and School of Medicine, National Yang-Ming University, Taipei, Taiwan; 5 Division of Infectious Diseases, Department of Medicine, Taipei Veterans General Hospital, Taipei, Taiwan; 6 Department of Family Medicine, Taipei Veterans General Hospital, Taipei, Taiwan; 7 University of Birmingham Institute of Cardiovascular Sciences, City Hospital, Birmingham, United Kingdom

## Abstract

Sudden cardiac death (SCD) is the most devastating manifestation of ventricular arrhythmias (VAs), and is the leading cause of mortality among atrial fibrillation (AF) patients. The goal of the present study was to investigate the incidence of SCD/VAs amongst patients with and without AF. We also aimed to identify important risk factors of SCD/VAs among AF patients. Using the “National Health Insurance Research Database” in Taiwan, a total of 352,656 AF and 352,656 non-AF patients without antecedent SCD/VAs were identified. The annual risk of SCD/VAs was higher in AF than non-AF groups (0.97% versus 0.47%) with an adjusted hazard ratio (HR) of 1.64. The increased risk of SCD/VAs in AF patients was consistently observed in different age strata, various comorbidities and patients without use of class I/III anti-arrhythmic drugs or digoxin. Among AF patients, age ≥75 years, congestive heart failure, hypertension, diabetes mellitus, previous stroke/transient ischemic attack, vascular diseases, chronic kidney disease and chronic obstructive pulmonary disease were important risk factors for SCD/VAs. In conclusion, the risk of SCD/VAs amongst AF patients was 1.64-fold higher compared to non-AF patients, which was associated with the number of clinical risk factors associated with the particular AF patient.

Atrial fibrillation (AF) is the most common sustained cardiac arrhythmia, and its prevalence is projected to continuously increase over the next few decades^1^,^2^. AF not only increases the risk of stroke, but also increases the risk of death by 1.5 fold in males and 1.9-fold in females in Framingham Heart Study^3^. Sudden cardiac death (SCD), the most devastating manifestation of ventricular arrhythmias (VAs), including ventricular tachycardia and ventricular fibrillation, was the leading cause of mortality (around 22%) among AF patients enrolled in the Randomized Evaluation of Long-Term Anti-coagulation Therapy (RE-LY) trial^4^. A recent study analyzing the data from the Atherosclerosis Risk in Communities (ARIC) and Cardiovascular Health Study (CHS) cohorts has demonstrated that the risk of SCD was higher in AF patients (n = 2,352) than non-AF patients (n = 18,566) with a hazard ratio (HR) of 2.47 after the adjustment for multiple confounders^5^.

However, the relationship between the risk of SCD/VAs and AF has not been well studied in a “non-selected” nationwide cohort. Furthermore, the incidence of SCD/VAs stratified by age, gender and its predictors remains unclear, especially in an Asian cohort of AF patients. The goal of the present study was to investigate the incidence of SCD/VAs of AF patients, and compare their risk to non-AF subjects in relation to gender and age strata. Second, we also aimed to identify important risk factors for SCD/VAs among AF patients.

## Methods

Nearly all Taiwanese residents were covered by the National Health Insurance (NHI) system, which offers comprehensive medical care coverage. The “National Health Insurance Research Database (NHIRD)” which included detailed information about health care of more than 23 million enrollees was released by the Taiwan government for the research purpose. The large sample size of this database provides a good opportunity to study the incidence and risk factors for SCD/VAs among AF patients.

### Study cohort

**Figure 1:**
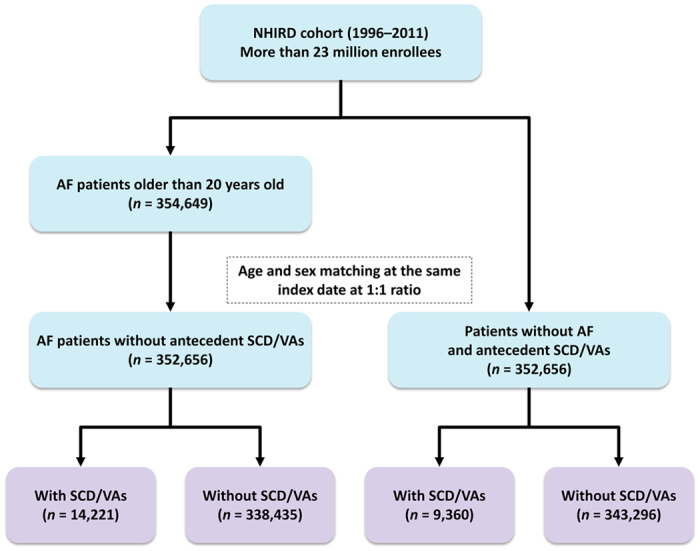
A flowchart of the enrollment of the study cohort. A total of 352,656 newly-diagnosed AF patients without history of SCD/VAs were identified from Taiwan NHIRD. One patient without AF and history of SCD/VAs matched for age and gender for each AF patient was selected as the control group. During the follow up, there were 14,221 and 9,360 patients who experienced SCD/VAs among the AF and non-AF groups, respectively. AF = atrial fibrillation; NHIRD = National Health Insurance Research Database; SCD = sudden cardiac death; VAs = ventricular arrhythmias.

The study design of the present study is similar to our previous studies using this dataset^6^,^7^,^8^,^9^,^10^. Over the 16-year period, 1996 to 2011, 354,649 newly-diagnosed AF patients aged ≥20 years were identified from the NHIRD as the study population. AF was diagnosed using the International Classification of Diseases (ICD), Ninth Revision, Clinical Modification (ICD-9-CM) codes (427.31). Patients were diagnosed to have AF only when it was a discharge diagnosis or was confirmed on at least two occasions in the outpatient department. The diagnostic accuracy of AF with this definition in Taiwan NHIRD has been validated in previous studies^11^,^12^. Among the study population, we excluded patients who have experienced and survived from previous episodes of SCD/VAs. Finally, a total of 352,656 AF patients without antecedent SCD/VAs were identified as the study population. For each AF patient, 1 age- and gender-matched control without AF and history of SCD/VAs was selected to constitute the control group (n = 352,656). Figure 1 shows the flowchart of the enrollment of the study cohort.

The comorbidities of each subject were diagnosed according to the ICD-9-CM codes registered by clinical physicians responsible for the care of the patient. Previous studies have validated the diagnostic accuracies of important systemic diseases in Taiwan NHIRD, including myocardial infarction, congestive heart failure, diabetes mellitus, ischemic stroke, hyperlipidemia, chronic obstructive pulmonary disease (COPD) and hypertension^13^,^14^,^15^. Insurance premiums, calculated according to the beneficiary’s total income, were used to estimate monthly income. Monthly income was grouped into low income (monthly income <20,000 New Taiwan Dollar [NTD]), medium income (20,000 NTD ≤monthly income <40,000 NTD), and high income (monthly income ≥40,000 NTD)^16^. Information about the degree of urbanization (urban, suburban or rural) of each patient was available in Taiwan NHIRD based on the townships where the patients lived. The stratifications of townships were based on the township population density (people/km^2^), population ratio of people with college or above educational levels, population ratio of elder people over 65 years old, population ratio of people of agriculture workers and the number of physicians per 100,000 people (http://ntur.lib.ntu.edu.tw/bitstream/246246/176519/1/5.pdf).

### Clinical endpoints

The clinical endpoints were defined as hospitalization or emergency department visit with the principal diagnosis of SCD (ICD-9-CM code 427.5, 798.1, or 798.2) and VAs (including ventricular tachycardia [ICD-9-CM code 427.1] and ventricular fibrillation and flutter [ICD-9-CM code 427.4, 427.41, or 427.42])^17^,^18^. During the mean follow up of 4.87 ± 3.97 years, there were 14,221 and 9,360 patients who experienced SCD/VAs in the AF and non-AF groups, respectively (Fig. 1).

### Statistical analysis

Continuous variables were summarized using mean value and standard deviation, and categorical variables were summarized using frequency and percentage. The differences between continuous variables and nominal variables were assessed with unpaired two-tailed t-test and chi-squared test, respectively. Incidence rates of SCD/VAs were calculated from dividing the number of event by person-time at risk. The cumulative incidence curve of SCD/VAs was plotted via the Kaplan-Meier method, with statistical significance examined by the log-rank test. Independent predictors for the risk of SCD/VAs were assessed using a multivariable Cox regression analysis. All statistical significances were set at a *p* < 0.05.

The study was approved by the Institutional Review Board (IRB) at Taipei Veterans General Hospital, Taipei, Taiwan. Since the patients’ original identification numbers have been encrypted to protect their privacy in NHIRD, the IRB agreed that the inform consent of the patient could be waived.

## Results

Baseline characteristics of AF and non-AF patients are shown in Supplemental Table 1. Age and gender were matched between the 2 groups. AF patients had more comorbidities, except for vascular disease and end-stage renal disease (ESRD).

**Figure 2:**
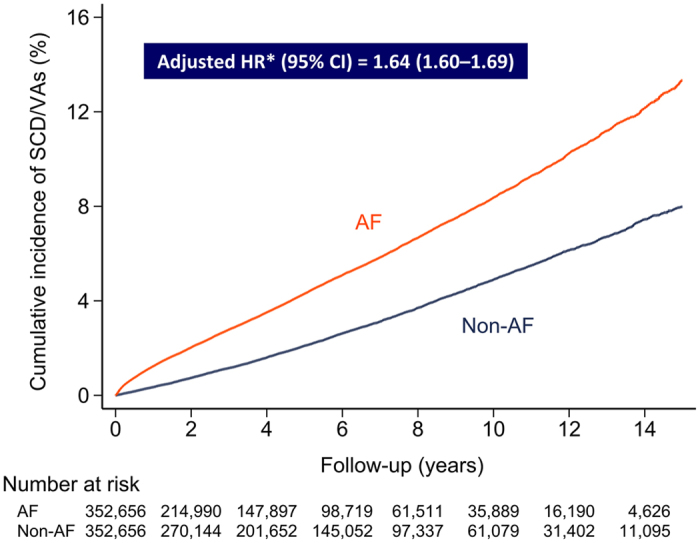
Cumulative incidence curves for SCD/VAs in AF and non-AF groups. The cumulative incidence curve with log rank test demonstrated that AF patients were associated with a higher risk of SCD/VAs than non-AF patients during the follow up period with an adjusted hazard ratio of 1.64 (95% CI = 1.60–1.69). *Adjusted for age, gender, comorbidities listed in Supplemental Table 1, degree of urbanization and income level. AF = atrial fibrillation; CI = confidence interval; SCD = sudden cardiac death; VAs = ventricular arrhythmias.

During the follow up of 3,437,771 person-years, 23,581 patients suffered from SCD/VAs (14,221 in AF and 9,360 in non-AF groups). Baseline characteristics of these patients are shown in Supplemental Table 2. The annual risk of SCD/VAs was higher in the AF group compared to the non-AF group (0.97% versus 0.47%). AF patients were associated with a higher risk of SCD/VAs than non-AF patients during the follow up period, with an adjusted hazard ratio (HR) of 1.64 (95% CI 1.60–1.69) (Fig. 2). AF patients were still associated with an increased risk of SCD/VAs with an adjusted HR of 1.72 (95% CI 1.67–1.77, p < 0.001) after considering age, sex and important comorbidities in the competing-risk model.

**Figure 3:**
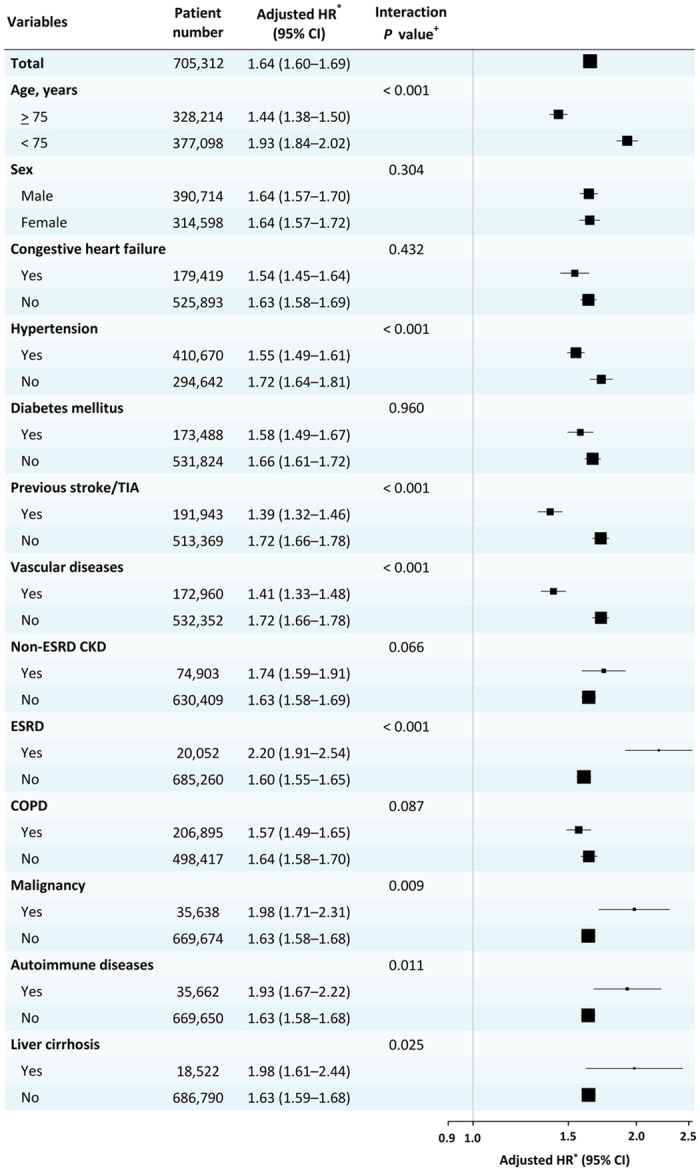
AF and risk of SCD/VAs in different subgroups of patients. In the subgroup analyses, AF was consistently associated with a higher risk of SCD/VAs compared to non-AF patients. *+Adjusted for age, gender, comorbidities listed in Supplemental Table 1, degree of urbanization and income level. AF = atrial fibrillation; CI = confidence interval; CKD = chronic kidney disease; COPD = chronic obstructive pulmonary disease; ESRD = end-stage renal disease; HR = hazard ratio; SCD = sudden cardiac death; SD = standard deviation; TIA = transient ischemic attack; VAs = ventricular arrhythmias.

In subgroup analyses, AF was consistently associated with a higher risk of SCD/VAs compared to non-AF patients in different subgroups of patients (Fig. 3). The associated risk was particularly higher amongst those age <75 (p_int_ < 0.001), ESRD (p_int_ < 0.001), malignancy (p_int_ = 0.009), autoimmune disease (p_int_ = 0.011), liver cirrhosis (p_int_ = 0.025) and those without hypertension (p_int_ < 0.001), previous stroke/transient ischemic attack (p_int_ < 0.001), and vascular diseases (p_int_ < 0.001). The risk of SCD/VAs was still higher among AF patients without use of class I/III anti-arrhythmic drugs (AADs) and digoxin (n = 252,843) compared to non-AF patients with an adjusted HR of 1.66 (95% CI 1.60–1.72).

### Risk of SCD/VAs in AF and non-AF patients stratified by age and gender

**Figure 4:**
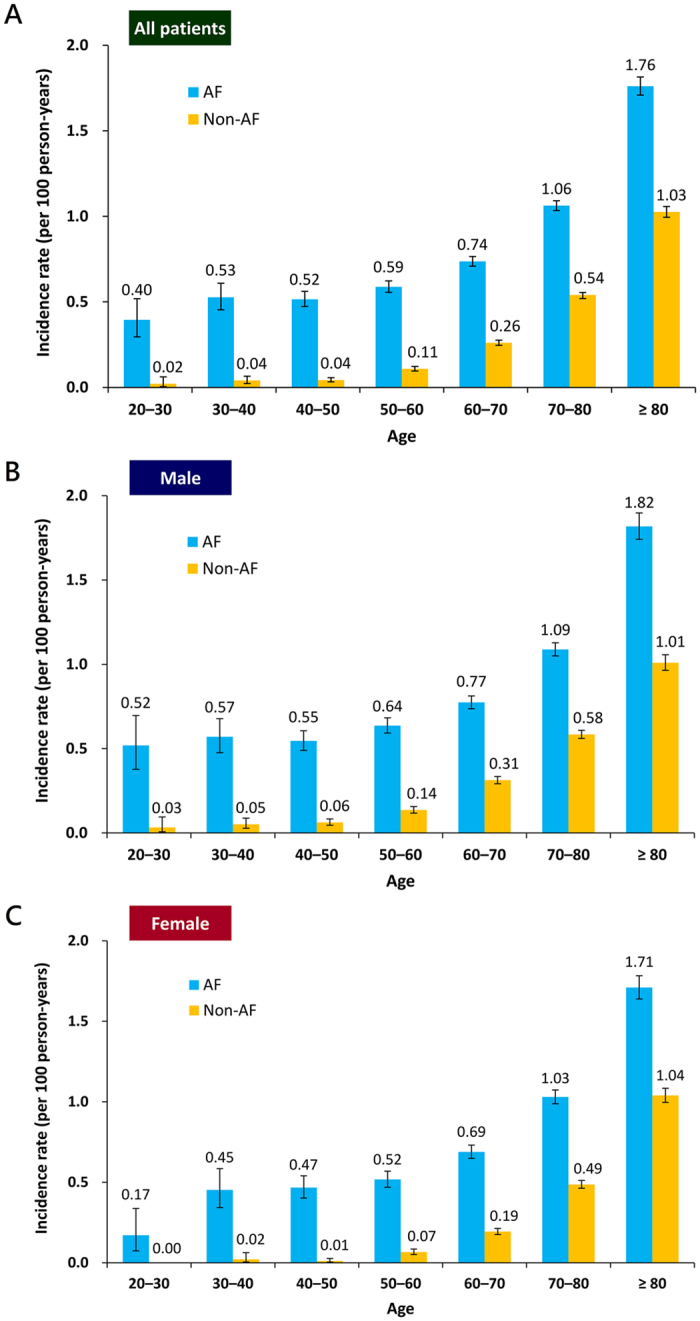
Risk of SCD/VAs in AF and non-AF patients stratified by age and gender. The risk of SCD/VAs was higher in AF than non-AF patients in each age strata. AF = atrial fibrillation; SCD = sudden cardiac death; VAs = ventricular arrhythmias.

**Figure 5:**
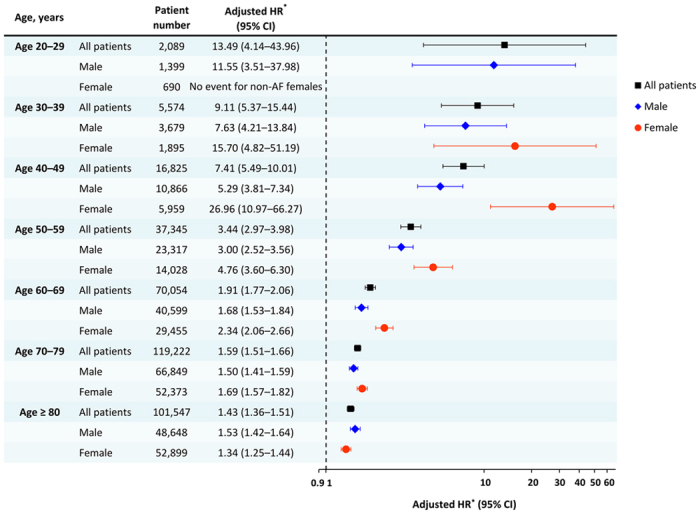
Risk of SCD/VAs of AF compared to non-AF patients represented by hazard ratios. AF was an important risk factor of SCD/VAs cross all age stratum in each gender. *Adjusted for age, gender, comorbidities listed in Supplemental Table 1, degree of urbanization and income level. AF = atrial fibrillation; CI = confidence interval; HR = hazard ratio; SCD = sudden cardiac death; VAs = ventricular arrhythmias.

Figure 4 shows the annual risk of SCD/VAs of AF and non-AF patients stratified by age for all (Fig. 4A), male (Fig. 4B) and female (Fig. 4C) patients. The risk of SCD/VAs continuously increased with age, and was consistently higher in AF patients in each age strata irrespective of gender. Figure 5 demonstrates the risk of SCD/VAs of AF compared to non-AF patients represented by HRs. AF significantly increased the risk of SCD/VAs in all age groups irrespective of gender, especially amongst younger patients.

### Propensity-matched analysis

Because AF and non-AF patients have different baseline characteristics, we performed a propensity score–matched analysis, in which we calculated a propensity score for the likelihood of AF by multivariate logistic regression analysis, conditional on the baseline covariates listed in Supplemental Table 1. After that, we matched each AF patient to a non-AF patient on the basis of propensity score (±0.01). Baseline characteristics of the AF and non-AF patients after the propensity match were shown in Supplemental Table 3. The propensity scores were similar between 2 groups (0.53 versus 0.53, p = 0.175). The annual risk of SCD/VAs was higher in the AF group compared to the non-AF group (0.96% versus 0.45%). AF patients were associated with a higher risk of SCD/VAs than non-AF patients with a HR of 1.52 (95% CI 1.49–1.57).

### Risk factors for SCD/VAs among AF patients

Among 352,656 AF patients with a follow-up of 4.15 ± 3.78 years, 14,221 patients (4.2%) experienced SCD/VAs. The baseline characteristics of AF patients with or without SCD/VAs are shown in Table 1. AF patients who suffered from SCD/VAs were older with a higher prevalence rate of comorbidities, except for malignancy, autoimmune diseases and liver cirrhosis, compared to patients without SCD/VAs.

**Figure 6:**
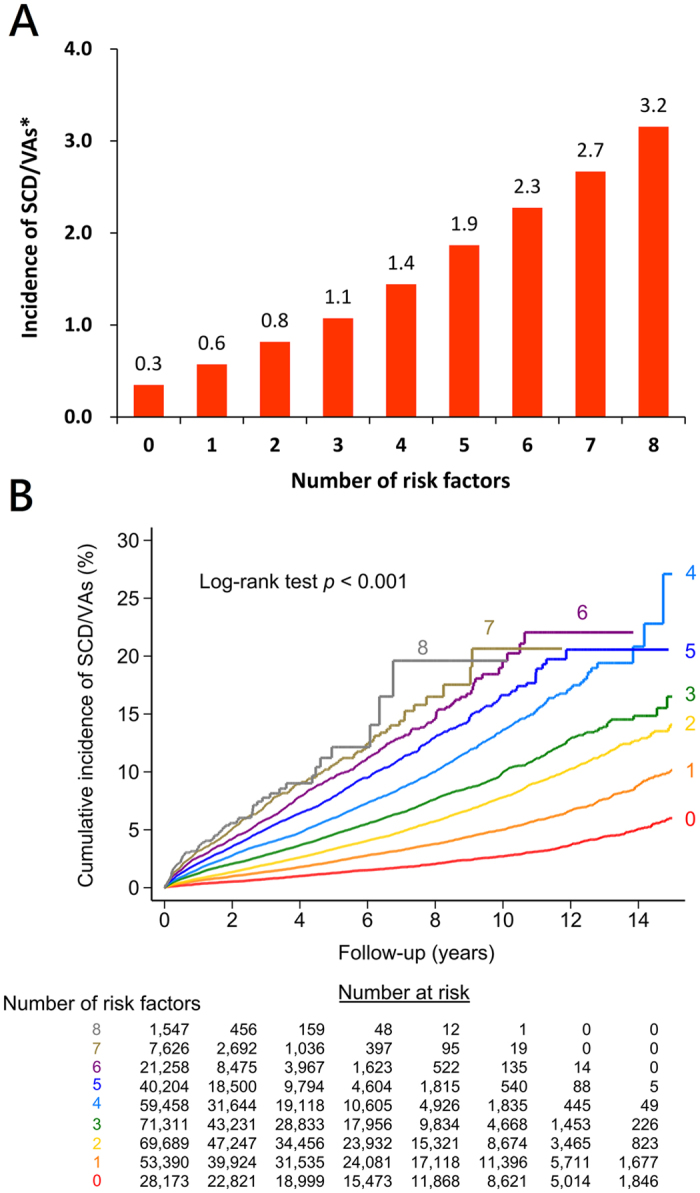
Incidence (**A**) and cumulative event rate (**B**) of SCD/VAs in AF patients with multiple risk factors. (**A**) The incidence of SCD/VAs continuously increased in patients with multiple risk factors. (**B**) Patients were stratified into 9 groups based on the number of risk factors they had. The cumulative incidence curve with log rank test showed that patients with more risk factors had a higher rate of SCD/VAs. *Number of SCD/VAs per 100 person-years of follow-up. AF = atrial fibrillation; CI = confidence interval; HR = hazard ratio; SCD = sudden cardiac death; VAs = ventricular arrhythmias.

Among the AF patients, age ≧75 years, congestive heart failure, hypertension, diabetes mellitus, previous stroke/transient ischemic attack (TIA), vascular diseases, chronic kidney disease (CKD), including ESRD and non-ESRD CKD, and COPD were identified to be independent risk factors for SCD/VAs (Table 2). The risk of SCD/VAs was proportional to the number of risk factors attributable to a patient (Fig. 6A), where the HR of SCD/VAs increased from 1.68 (95% CI 1.54–1.84) for 1 risk factor rising to 8.75 (95% CI 6.92–11.08) for patients with 8 risk factors, in comparison to those without any risk factors (Supplemental Table 4).

With an increase in the number of risk factors in AF patients, the cumulative event rate correspondingly increased (Fig. 6A and B). Even for AF patients without any significant clinical risk factors, the annual risk of SCD/VAs was around 0.3%, which was still higher than non-AF patients with an adjusted HR of 2.03 (95% CI 1.85–2.23, p value < 0.001).

## Discussion

In this large-scale natiowide cohort study, we our principal findings are as follows: (1) The annual risk of SCD/VAs of AF patients was around 0.97%, which was 1.64-fold higher than non-AF patients; (2) The increased risk of SCD/VAs in AF patients was consistently observed in differernt age strata, various comorbidities and patients without use of class I/III AADs or digoxin; and (3) The individual risk of SCD/VAs for AF patients can be estimated according to the number of clinical risk factors including age ≥75 years, congestive heart failure, hypertension, diabetes mellitus, previous stroke/TIA, vascular diseases, CKD and COPD.

### Risk of SCD/VAs in AF

Evidence suggesting that AF may increase the risk of VAs comes from recipients of implantable cardioverter-defibrillators (ICDs). For example, in one prospective study which enrolled 250 patients receiving ICD for secondary prevention, AF patients experienced appropriate device therapy for recurrent VAs 1.8 fold more commonly, compared with non-AF patients^19^. An analysis of device-stored electrograms revealed a higher incidence of short-long-short cycles preceding VAs in AF compared to non-AF patients (55% vs 31%, p = 0.01). The relationship between new-onset AF and risk of SCD was also been evaluated in 8,831 hypertensive patients with electrocardiographic left ventricular hypertrophy without history of AF enrolled in the Losartan Intervention For Endpoint reduction (LIFE) study^20^. After adjustment for potential confounders, AF remained as an important risk factor of SCD with a HR 3.13 (95% CI 1.87–5.24). In a recent study which analyzed data from ARIC study and CHS, a significant link between AF and SCD was been demonstrated among community-dewelling individuals^5^.

Although the association between AF and SCD/VAs has been reported, the risk of SCD/VAs for AF patients stratified by age and gender remains unknown due to the selected patient population in previous studies with a small sample sizes. In the present study, the mean age of AF population was 74 years which was similar to that of patients enrolled in CHS (73 years), and the annual risks of SCD/VAs were also similar (0.97% versus 1.2%)^5^. Of particular importance, the present study is the first large-scale nationwide cohort study providing the epidemiological data regarding the risk of SCD/VAs, specifically among Asian AF patients with data for gender and each age strata. For both AF and non-AF patients, the risk of SCD/VAs continuously increased with age, and the risk of SCD/VAs of AF patients age <40 years was ten-fold higher than non-AF controls. The AF-associated increase in risk of SCD/VAs was higher for patients age <75 years than those aged ≥75 years (HR = 1.93 versus 1.44, p_int_ < 0.001, Fig. 3). Although the HRs of SCD/VAs in AF were attenuated among patients aged ≥75 years, the risk still remained statistically significant (HR = 1.44, 95% CI = 1.38–1.50, Fig. 3).

While we have demonstrated that AF patients were associated with an increased risk of SCD/VAs, the detailed mechanisms behind the link were unclear. It is possible that the association between AF and SCD is mediated by shared risk factors such as heart failure and vascular diseases. However, AF remained significantly associated with SCD/VAs after the adjustments for potential confounders using multivariate Cox regression, propensity-matched and competing-risk analyses in the present study. Besides, AF-associated increases in risk of SCD/VAs are statistically similar for patients with or without heart failure with HRs of 1.54 and 1.63, respectively (p_int_ = 0.432). In the previous analysis of ARIC cohort, the risk of SCD was still higher for AF patients after the adjustment for fractional shortening of left ventricle measured by 2-dimensional echocardiogram^5^. These results may suggest that the association between AF and SCD/VAs is not simply mediated by left ventricular dysfunction, and could not be completely explained by shared risk factors. Indeed, a rapid ventricular rate during an atrial tachyarrhythmia could reduce ventricular refractoriness and promote VAs^21^. In addition, the irregular rhythm of AF may lead to short-long-short sequences which are intrinsically proarrhythmic^22^. Therefore, AF itself may facilitate the induction of VAs and cause SCD^23^.

### Clinical implications

Although the risk of SCD/VAs is higher in AF than non-AF subjects, the risk factors accentuating this risk and how to estimate the individual risk of SCD/VAs for AF patients were not been well investigated. In the present study, several important clinical factors associated with the occurrence of SCD/VAs were identified. We found that the incidence of SCD/VAs increased as more risk factors were present, increasing to 3.2%/year for AF patients with all 8 risk factors, constituting a 8.75 fold increased risk for the development of SCD/VAs in comparison with patients without any risk factors. The annual risk of SCD/VAs for each AF patient could be easily estimated based on the number of the risk factors that the patient has. Thus, clinicians should pay attention to the potential risk of SCD/VAs for AF patients with multiple risk factors, especially when patients experience episodes of near fainting or syncope. Whether more aggressive treatment and better control of blood pressure, hyperglycemia and congestive heart failure could decrease the risk of SCD/VAs deserves further study.

### Limitations

First, since AF patients had more comorbidities than non-AF patients, whether AF was the direct cause responsible for the higher risk of SCD/VAs remains unknown. Although we have tried to adjust for age, sex and important comorbidities in the competing-risk model, we were not able to exclude the possibility that some other confounders were not recognized and may therefore confound the analysis. Second, data on the subtypes of AF were not available, and therefore we can not investigate whether the risk of SCD/VAs were different for paroxysmal and non-paroxysmal AF patients. Third, the diagnosis of SCD/VAs was based on the diagnostic codes registered in the database, and the real incidence of SCD/VAs is likely to be underestimated. Besides, we were not able to further clarify the SCD event as being witnessed or unwitnessed. Fourth, age is a continuous variable which was associated with an increased risk of SCD/VAs, and the setting of the cutoff value (75 years) is just for the convenience to assess patient’s risk in the daily practice. Indeed, age 65–74 years was also associated with an increased risk of SCD/VA with an adjusted HR of 1.27 (95% CI = 1.21–1.33) compared to patients aged <65 years. However, the HR of SCD/VA associated with an age ≥75 years (1.63, 95% CI = 1.57–1.69) was higher than that of age 65–74 years. Last, the present study only enrolled Asian (ie. Taiwanese) patients, and whether the results can be extrapolated to other populations remains uncertain.

## Conclusion

In this nationwide cohort study, the risk of SCD/VAs amongst AF patients was 1.64-fold higher compared to non-AF patients. The individual risk of SCD/VAs can be associated with the number of clinical risk factors associated with the particular AF patient.

## Additional Information

**How to cite this article**: Chao, T.-F. *et al*. Risk and Prediction of Sudden Cardiac Death and Ventricular Arrhythmias for Patients with Atrial Fibrillation – A Nationwide Cohort Study. *Sci. Rep.* **7**, 46445; doi: 10.1038/srep46445 (2017).

**Publisher's note:** Springer Nature remains neutral with regard to jurisdictional claims in published maps and institutional affiliations.

## Supplementary Material

Supplementary Tables

## Figures and Tables

**Table 1:** Baseline characteristics of AF patients with or without SCD/VAs.

Variables	With SCD/VAs (*n* = 14,221)	Without SCD/VAs (*n* = 338,435)	*P* value
Age, years; mean value (SD)	72.4 (12.2)	71.3 (13.1)	<0.001
Age ≥75 years, *n* (%)	7,038 (49.5)	157,069 (46.4)	<0.001
Male gender, *n* (%)	8,000 (56.3)	187,357 (55.4)	0.035
Comorbidities, *n* (%)			
Congestive heart failure	7,291 (51.3)	136,621 (40.4)	<0.001
Hypertension	10,121 (71.2)	231,210 (68.3)	<0.001
Diabetes mellitus	4,619 (32.5)	96,065 (28.4)	<0.001
Previous stroke/TIA	5,175 (36.4)	117,707 (34.8)	<0.001
Vascular diseases	3,421 (24.1)	76,125 (22.5)	<0.001
Non-ESRD CKD	2,426 (17.1)	51,157 (15.1)	<0.001
ESRD	495 (3.5)	7,261 (2.2)	<0.001
COPD	5,368 (37.8)	119,690 (35.4)	<0.001
Malignancy	563 (4.0)	18,857 (5.6)	<0.001
Autoimmune diseases	700 (4.9)	18,766 (5.5)	0.001
Liver cirrhosis	428 (3.0)	11,356 (3.4)	0.025
Medications, *n* (%)			
Aspirin	4,513 (31.7)	102,647 (30.3)	<0.001
Clopidogrel	679 (4.8)	13,882 (4.1)	<0.001
Warfarin	1,664 (11.7)	40,222 (11.9)	0.507
Beta-blockers	2,234 (15.7)	60,050 (17.7)	<0.001
CCBs	1,374 (9.7)	30,267 (8.9)	0.003
Digoxin	3,205 (22.5)	57,915 (17.1)	<0.001
Class I AADs	427 (3.0)	12,388 (3.7)	<0.001
Class III AADs	1,359 (9.6)	29,650 (8.8)	0.001
ACEIs/ARBs	4,344 (30.6)	97,110 (28.7)	<0.001
Statins	712 (5.0)	13,948 (4.1)	<0.001
Degree of urbanization, n (%)			
Urban	7,498 (52.7)	174,095 (51.4)	0.004
Suburban	4,658 (32.8)	112,536 (33.3)	
Rural	2,065 (14.5)	51,804 (15.3)	
Income level, *n* (%)			
Low	7,748 (54.5)	181,336 (53.6)	0.031
Median	4,696 (33.0)	112,537 (33.3)	
High	1,777 (12.5)	44,562 (13.2)	

AADs = anti-arrhythmic drugs; ACEI = angiotensin-converting enzyme inhibitor; AF = atrial fibrillation; ARB = angiotensin II-receptor blocker; CCBs = calcium channel blockers; CKD = chronic kidney disease; COPD = chronic obstructive pulmonary disease; ESRD = end-stage renal disease; SD = standard deviation; TIA = transient ischemic attack.

**Table 2:** Clinical predictors of SCD/VAs among AF patients.

Variables	Univariate analysis		Multivariate analysis*	
	HR (95% CI)	*P* value	HR (95% CI)	*P* value
Age ≥75 years	1.97 (1.91–2.04)	<0.001	1.63 (1.57–1.69)	<0.001
Male gender	1.02 (0.99–1.05)	0.251		
Comorbidities				
Congestive heart failure	2.01 (1.94–2.07)	<0.001	1.69 (1.63–1.75)	<0.001
Hypertension	1.51 (1.46–1.57)	<0.001	1.14 (1.10–1.19)	<0.001
Diabetes mellitus	1.61 (1.56–1.67)	<0.001	1.35 (1.30–1.40)	<0.001
Previous stroke/TIA	1.51 (1.46–1.57)	<0.001	1.24 (1.19–1.28)	<0.001
Vascular diseases	1.22 (1.18–1.27)	<0.001	1.06 (1.02–1.11)	0.004
Non-ESRD CKD	1.72 (1.64–1.80)	<0.001	1.32 (1.26–1.38)	<0.001
ESRD	2.70 (2.47–2.95)	<0.001	2.49 (2.27–2.73)	<0.001
COPD	1.60 (1.54–1.65)	<0.001	1.21 (1.16–1.25)	<0.001
Malignancy	1.17 (1.08–1.28)	<0.001	1.05 (0.96–1.14)	0.281
Autoimmune diseases	1.13 (1.05–1.22)	0.002	0.93 (0.86–1.00)	0.050
Liver cirrhosis	1.27 (1.15–1.40)	<0.001	1.08 (0.98–1.19)	0.116

*Variables with p values < 0.05 in the univariate analyses were included in the multivariate model which was adjusted for the use of medications listed in Table 1, degree of urbanization and income level. AF = atrial fibrillation; CI = confidence interval; CKD = chronic kidney disease; COPD = chronic obstructive pulmonary disease; ESRD = end-stage renal disease; HR = hazard ratio; SCD = sudden cardiac death; TIA = transient ischemic attack; VAs = ventricular arrhythmias.

## References

[b1] Miyasaka Y. . Secular trends in incidence of atrial fibrillation in Olmsted County, Minnesota, 1980 to 2000, and implications on the projections for future prevalence. Circulation 114, 119–125 (2006). 16818816 10.1161/CIRCULATIONAHA.105.595140

[b2] Tse H. F. . Stroke prevention in atrial fibrillation–an Asian stroke perspective. Heart Rhythm 10, 1082–1088 (2013). 23501173 10.1016/j.hrthm.2013.03.017

[b3] Benjamin E. J. . Impact of atrial fibrillation on the risk of death: the Framingham Heart Study. Circulation 98, 946–952 (1998). 9737513 10.1161/01.cir.98.10.946

[b4] Marijon E. . Causes of death and influencing factors in patients with atrial fibrillation: a competing-risk analysis from the randomized evaluation of long-term anticoagulant therapy study. Circulation 128, 2192–2201 (2013). 24016454 10.1161/CIRCULATIONAHA.112.000491

[b5] Chen L. Y. . Atrial fibrillation and the risk of sudden cardiac death: the atherosclerosis risk in communities study and cardiovascular health study. JAMA internal medicine 173, 29–35 (2013). 23404043 10.1001/2013.jamainternmed.744 PMC3578214

[b6] Chao T. F. . Acute myocardial infarction in patients with atrial fibrillation with a CHA2DS2-VASc score of 0 or 1: a nationwide cohort study. Heart Rhythm 11, 1941–1947 (2014). 25101483 10.1016/j.hrthm.2014.08.003

[b7] Chao T. F. . Does digoxin increase the risk of ischemic stroke and mortality in atrial fibrillation? A nationwide population-based cohort study. Can J Cardiol 30, 1190–1195 (2014). 25262860 10.1016/j.cjca.2014.05.009

[b8] Chao T. F. . Using the CHA2DS2-VASc score for refining stroke risk stratification in ‘low-risk’ Asian patients with atrial fibrillation. J Am Coll Cardiol 64, 1658–1665 (2014). 25323252 10.1016/j.jacc.2014.06.1203

[b9] Chao T. F. . Should atrial fibrillation patients with 1 additional risk factor of the CHA2DS2-VASc score (beyond sex) receive oral anticoagulation? J Am Coll Cardiol 65, 635–642 (2015). 25677422 10.1016/j.jacc.2014.11.046

[b10] Liao J. N. . Risk and prediction of dementia in patients with atrial fibrillation - A nationwide population-based cohort study. Int J Cardiol 199, 25–30 (2015). 26173170 10.1016/j.ijcard.2015.06.170

[b11] Lin L. J. . Compliance with antithrombotic prescribing guidelines for patients with atrial fibrillation–a nationwide descriptive study in Taiwan. Clin Ther 30, 1726–1736 (2008). 18840379 10.1016/j.clinthera.2008.09.010

[b12] Chang C. H. . Continuation of statin therapy and a decreased risk of atrial fibrillation/flutter in patients with and without chronic kidney disease. Atherosclerosis 232, 224–230 (2014). 24401243 10.1016/j.atherosclerosis.2013.11.036

[b13] Lin C. C., Lai M. S., Syu C. Y., Chang S. C. & Tseng F. Y. Accuracy of diabetes diagnosis in health insurance claims data in Taiwan. Journal of the Formosan Medical Association 104, 157–163 (2005). 15818428

[b14] Cheng C. L., Kao Y. H., Lin S. J., Lee C. H. & Lai M. L. Validation of the National Health Insurance Research Database with ischemic stroke cases in Taiwan. Pharmacoepidemiol Drug Saf 20, 236–242 (2011). 21351304 10.1002/pds.2087

[b15] Hsieh C. Y., Chen C. H., Li C. Y. & Lai M. L. Validating the diagnosis of acute ischemic stroke in a National Health Insurance claims database. Journal of the Formosan Medical Association 114, 254–259 (2015). 24140108 10.1016/j.jfma.2013.09.009

[b16] Perng C. L. . Risk of depressive disorder following non-alcoholic cirrhosis: a nationwide population-based study. PloS one 9, e88721 (2014). 24533141 10.1371/journal.pone.0088721 PMC3922987

[b17] Shih C. J. . Long-term clinical outcome of major adverse cardiac events in survivors of infective endocarditis: a nationwide population-based study. Circulation 130, 1684–1691 (2014). 25223982 10.1161/CIRCULATIONAHA.114.012717

[b18] Wu C. S., Tsai Y. T. & Tsai H. J. Antipsychotic drugs and the risk of ventricular arrhythmia and/or sudden cardiac death: a nation-wide case-crossover study. Journal of the American Heart Association 4, e001568, doi: 10.1161/JAHA.114.001568 (2015). 25713294 PMC4345877

[b19] Gronefeld G. C., Mauss O., Li Y. G., Klingenheben T. & Hohnloser S. H. Association between atrial fibrillation and appropriate implantable cardioverter defibrillator therapy: results from a prospective study. J Cardiovasc Electrophysiol 11, 1208–1214 (2000). 11083241 10.1046/j.1540-8167.2000.01208.x

[b20] Okin P. M. . Relationship of sudden cardiac death to new-onset atrial fibrillation in hypertensive patients with left ventricular hypertrophy. Circ Arrhythm Electrophysiol 6, 243–251 (2013). 23403268 10.1161/CIRCEP.112.977777

[b21] Denes P., Wu D., Dhingra R., Pietras R. J. & Rosen K. M. The effects of cycle length on cardiac refractory periods in man. Circulation 49, 32–41(1974). 4271710 10.1161/01.cir.49.1.32

[b22] Denker S., Lehmann M., Mahmud R., Gilbert C. & Akhtar M. Facilitation of ventricular tachycardia induction with abrupt changes in ventricular cycle length. Am J Cardiol 53, 508–515 (1984). 6198893 10.1016/0002-9149(84)90022-5

[b23] Chen L. Y., Benditt D. G. & Alonso A. Atrial fibrillation and its association with sudden cardiac death. Circ J 78, 2588–2593 (2014). 25262841 10.1253/circj.cj-14-0814

