# Supplementary material for: Risk and Prediction of Sudden Cardiac Death and Ventricular Arrhythmias for Patients with Atrial Fibrillation – A Nationwide Cohort Study
Source: Sci Rep. 2017 Apr 19;7:46445. doi: 10.1038/srep46445 (PMC5396069; doi:10.1038/srep46445)
Supplement: Supplementary Tables [file srep46445-s1.doc]

**Risk and Prediction of Sudden Cardiac Death and Ventricular Arrhythmias for Patients with Atrial Fibrillation – A Nationwide Cohort Study**

Tze-Fan Chao, M.D.^1,2^, Chia-Jen Liu, M.D.^3,4^, Ta-Chuan Tuan, M.D.^1,2^*****, Su-Jung Chen, M.D.^4,5^, Tzeng-Ji Chen, M.D.^6^, Gregory Y. H. Lip, M.D.^7^*****, and Shih-Ann Chen, M.D.^1,2^

^1^Division of Cardiology, Department of Medicine, Taipei Veterans General Hospital, Taipei, Taiwan. ^2^Institute of Clinical Medicine, and Cardiovascular Research Center, National Yang-Ming University, Taipei, Taiwan. ^3^Division of Hematology and Oncology, Department of Medicine, Taipei Veterans General Hospital, Taipei, Taiwan. ^4^Institute of Public Health and School of Medicine, National Yang-Ming University, Taipei, Taiwan. ^5^Division of Infectious Diseases, Department of Medicine, Taipei Veterans General Hospital, Taipei, Taiwan. ^6^Department of Family Medicine, Taipei Veterans General Hospital, Taipei, Taiwan. ^7^University of Birmingham Institute of Cardiovascular Sciences, City Hospital, Birmingham, United Kingdom

**Dr. Tze-Fan Chao and Dr. Chia-Jen Liu contributed equally to this study**

**[* Joint senior authors]**

Running title: Risk and prediction of SCD/VAs in AF

**Address for correspondence**

**Ta-Chuan Tuan**, M.D.

Division of Cardiology, Department of Medicine, Taipei Veterans General Hospital,

No. 201, Sec. 2, Shih-Pai Road, Taipei, Taiwan.

Tel: 886-2-2875-7156

Fax: 886-2-2873-5656

E-Mail: duan.dachuan@gmail.com

**Supplemental** **Table 1 Baseline characteristics of patients with or without AF**

**(n = 705,312)**

| **Variables** | **AF**  **(*n* =** **352,656)** | **Non-AF**  **(*n* = 352,656)** | ***P* value** |
| --- | --- | --- | --- |
| Age, years; mean value (SD) | 74 (13.0) | 74 (13.0) | 1.000 |
| Age ≥ 75 years, *n* (%) | 164,107 (46.5) | 164,107 (46.5) | 1.000 |
| Male gender, *n* (%) | 195,357 (55.4) | 195,357 (55.4) | 1.000 |
| Comorbidities, *n* (%) |  |  |  |
| Congestive heart failure | 143,912 (40.8) | 35,507 (10.1) | < 0.001 |
| Hypertension | 241,331 (68.4) | 169,339 (48.0) | < 0.001 |
| Diabetes mellitus | 100,684 (28.6) | 72,804 (20.6) | < 0.001 |
| Previous stroke/TIA | 122,882 (34.8) | 69,061 (19.6) | < 0.001 |
| Vascular diseases | 79,546 (22.6) | 93,414 (26.5) | < 0.001 |
| Non-ESRD CKD | 53,583 (15.2) | 21,320 (6.1) | < 0.001 |
| ESRD | 7,756 (2.2) | 12,296 (3.5) | < 0.001 |
| COPD | 125,058 (35.5) | 81,837 (23.2) | < 0.001 |
| Malignancy | 19,420 (5.5) | 16,218 (4.6) | < 0.001 |
| Autoimmune diseases | 19,466 (5.5) | 16,196 (4.6) | < 0.001 |
| Liver cirrhosis | 11,784 (3.3) | 6,738 (1.9) | < 0.001 |
| Degree of urbanization, *n* (%) |  |  | < 0.001 |
| Urban | 181,593 (51.5) | 191,200 (54.2) |  |
| Suburban | 117,194 (33.2) | 109,783 (31.1) |  |
| Rural | 53,869 (15.3) | 51,673 (14.7) |  |
| Income level, *n* (%) |  |  | < 0.001 |
| Low | 189,084 (53.6) | 243,078 (68.9) |  |
| Median | 117,233 (33.2) | 66,998 (19.0) |  |
| High | 46,339 (13.1) | 42,580 (12.1) |  |

AF = atrial fibrillation; CKD = chronic kidney disease; COPD = chronic obstructive pulmonary disease; ESRD = end-stage renal disease; SD = standard deviation; TIA = transient ischemic attack

**Supplemental Table 2 Baseline characteristics of patients who experienced SCD/VAs** **with or without AF (n = 23,581)**

| **Variables** | **With SCD/VAs** | | ***P* value** |
| --- | --- | --- | --- |
|  | **AF**  **(*n* = 14,221)** | **Non-AF**  **(*n* = 9,360)** |  |
| Age, years; mean value (SD) | 72 (12.1) | 77 (9.0) | < 0.001 |
| Age ≥ 75 years, *n* (%) | 7,038 (49.5) | 6,175 (66.0) | < 0.001 |
| Male gender, *n* (%) | 8,000 (56.3) | 5198 (55.5) | 0.275 |
| Comorbidities, *n* (%) |  |  |  |
| Congestive heart failure | 7,291 (51.3) | 1,492 (15.9) | < 0.001 |
| Hypertension | 10,121 (71.2) | 4,966 (53.1) | < 0.001 |
| Diabetes mellitus | 4,619 (32.5) | 2,301 (24.6) | < 0.001 |
| Previous stroke/TIA | 5,179 (36.4) | 2,359 (27.1) | < 0.001 |
| Vascular diseases | 3,421 (36.4) | 3,024 (32.3) | < 0.001 |
| Non-ESRD CKD | 2,426 (17.1) | 633 (6.8) | < 0.001 |
| ESRD | 495 (3.5) | 522 (5.6) | < 0.001 |
| COPD | 5,368 (37.7) | 2,583 (27.6) | < 0.001 |
| Malignancy | 563 (4.0) | 313 (3.3) | 0.015 |
| Autoimmune diseases | 700 (4.9) | 331 (3.5) | < 0.001 |
| Liver cirrhosis | 428 (3.0) | 145 (1.5) | < 0.001 |
| Degree of urbanization, *n* (%) |  |  | 0.042 |
| Urban | 7,498 (52.7) | 5,075(54.2) |  |
| Suburban | 4,658 (32.8) | 2,969 (31.7) |  |
| Rural | 2,065 (14.5) | 1,316 (14.1) |  |
| Income level, *n* (%) |  |  | < 0.001 |
| Low | 7,748 (54.5) | 6,101 (65.2) |  |
| Median | 4,696 (33.0) | 2,117 (22.6) |  |
| High | 1,777 (12.5) | 1,442 (12.2) |  |

AF = atrial fibrillation; CKD = chronic kidney disease; COPD = chronic obstructive pulmonary disease; ESRD = end-stage renal disease; SD = standard deviation; TIA = transient ischemic attack

**Supplemental Table 3 Baseline characteristics of patients with or without AF** **after the propensity match (n =423,974)**

| **Variables** | **AF**  **(*n* = 211,987)** | **Non-AF**  **(*n* = 211,987)** | ***P* value** |
| --- | --- | --- | --- |
| Age, years; mean value (SD) | 74 (12.6) | 75 (11.5) | 0.895 |
| Male gender, *n* (%) | 114,556 (54.0) | 115,396 (54.4) | 0.011 |
| Comorbidities, *n* (%) |  |  |  |
| Congestive heart failure | 39,389 (18.6) | 35,493 (16.7) | < 0.001 |
| Hypertension | 129,001 (60.9) | 143,808 (67.8) | < 0.001 |
| Diabetes mellitus | 52,231 (24.6) | 56,333 (26.6) | < 0.001 |
| Previous stroke/TIA | 64,009 (30.2) | 64,315 (30.3) | 0.306 |
| Vascular diseases | 52,250 (24.6) | 58,449 (27.6) | < 0.001 |
| Non-ESRD CKD | 21,827 (10.3) | 20,305 (9.7) | 0.045 |
| ESRD | 5,300 (2.5) | 6,748 (3.2) | < 0.001 |
| COPD | 67,434 (31.8) | 38,444 (32.3) | 0.016 |
| Malignancy | 11,811 (5.6) | 12,668 (6.0) | 0.019 |
| Autoimmune diseases | 11,039 (5.2) | 11,351 (5.4) | 0.032 |
| Liver cirrhosis | 5,029 (2.4) | 5,539 (2.6) | 0.486 |
| Degree of urbanization, *n* (%) |  |  | 0.031 |
| Urban | 112,131 (53.0) | 113,213 (53.4) |  |
| Suburban | 69,425 (32.7) | 66,371 (31.3) |  |
| Rural | 30,430 (14.3) | 32,403 (15.3) |  |
| Income level, *n* (%) |  |  | < 0.001 |
| Low | 127,110 (60.0) | 130,883 (61.7) |  |
| Median | 63,448 (29.9) | 41,807 (19.7) |  |
| High | 21,429 (10.1) | 39,297 (18.5) |  |
| **Mean propensity score; mean (SD)** | **0.53 (0.18)** | **0.53 (0.18)** | **0.175** |

AF = atrial fibrillation; CKD = chronic kidney disease; COPD = chronic obstructive pulmonary disease; ESRD = end-stage renal disease; SD = standard deviation; TIA = transient ischemic attack

**Supplemental Table 4 Risk of SCD/VAs in AF patients with different number of risk factors**

| **Number**  **of risk factors** | **Number of**  **patient**  **(*n* = 352,656)** | **Number of**  **SCD/VAs**  **(*n* = 14,221)** | **Hazard ratio** | **95% CI** | ***P* value** |
| --- | --- | --- | --- | --- | --- |
| **0** | 28,173 | 681 | 1(reference) | - | - |
| **1** | 53,390 | 1,782 | 1.68 | 1.54–1.84 | < 0.001 |
| **2** | 69,689 | 2,694 | 2.46 | 2.26–2.67 | < 0.001 |
| **3** | 71,311 | 2,943 | 3.26 | 3.00–3.55 | < 0.001 |
| **4** | 59,458 | 2,692 | 4.38 | 4.02–4.77 | < 0.001 |
| **5** | 40,204 | 1,924 | 5.59 | 5.11–6.11 | < 0.001 |
| **6** | 21,258 | 1,046 | 6.67 | 6.04–7.36 | < 0.001 |
| **7** | 7,626 | 381 | 7.64 | 6.73–8.68 | < 0.001 |
| **8** | 1,547 | 78 | 8.75 | 6.92–11.08 | < 0.001 |

AF = atrial fibrillation; CI = confidence interval; SCD = sudden cardiac death; VAs = ventricular arrhythmias
